# The clinical and imaging presentation of acute "non complicated" pyelonephritis: A new profile for an ancient disease

**DOI:** 10.1186/1471-2369-12-68

**Published:** 2011-12-15

**Authors:** Giorgina Barbara Piccoli, Valentina Consiglio, Maria Chiara Deagostini, Melania Serra, Marilisa Biolcati, Francesca Ragni, Alberto Biglino, Agostino De Pascale, Mauro Felice Frascisco, Andrea Veltri, Francesco Porpiglia

**Affiliations:** 1 Nephrology; Department of Clinical and Biological Sciences ASOU san Luigi Gonzaga Regione Gonzole 10, Orbassano, University of Torino, Italy; 2 Urology; Department of Clinical and Biological Sciences ASOU san Luigi Gonzaga Regione Gonzole 10, Orbassano, University of Torino, Italy; 3 Radiology; Department of Clinical and Biological Sciences ASOU san Luigi Gonzaga Regione Gonzole 10, Orbassano, University of Torino, Italy; 4 Infectious Diseases Department of Clinical and Biological Sciences, Ospedale di Asti, University of Torino, Italy; 5 Emergency Medicine; Department of Clinical and Biological Sciences ASOU san Luigi Gonzaga Regione Gonzole 10, Orbassano, University of Torino, Italy; 6 Materno Foetal Unit, sant'Anna Hospital, University of Torino, Italy

**Keywords:** Acute pyelonephritis, upper urinary tract infections, computed tomography, nuclear magnetic resonance, kidney scars, epidemiology

## Abstract

**Background:**

Acute pyelonephritis (APN) is differently defined according to imaging or clinical criteria. In adults information on the relationship between imaging and clinical data is lacking.

Our study was aimed at analysing the relationship between the clinical and imaging presentation of APN, defined according to imaging criteria (parenchymal involvement at MR or CT scan).

**Methods:**

All consecutive patients hospitalized for "non-complicated" APN were considered (June 2005-December 2009). Clinical, biochemical and imaging data at hospitalization were analyzed by univariate and logistic regression analysis.

**Results:**

There were 119 patients, all females, median age 32 years (15-72). At hospitalization, inflammatory markers were elevated (CRP median: 12.1 mg/dL, normal < 0.8). Incomplete presentations were frequent: fever was absent in 6.7%, pain in 17.8%, lower urinary tract symptoms in 52.9%. At CT or MR scan the lesions were bilateral in 12.6%, multiple in 79.8%; abscesses were present in 39.5%. Renal scars were found in 15.1%. Positive cultures were correlated with multiple foci (multivariate OR 4.2; CI 1.139-15.515). No other sign/symptom discriminated between small lesions, abscesses or multifocal involvement.

**Conclusions:**

APN is a protean disease. In the absence of strict correlation with clinical or biochemical markers, imaging studies are required to assess the severity of kidney involvement.

## Background

Acute pyelonephritis (APN) is an extensively described, well-known disease. The first descriptions date to ancient Egypt, underlining its severity and its potential to lead to sepsis, kidney abscesses and destruction of the kidney parenchyma [1]. In spite of this long history, the nomenclature of acute pyelonephritis is still controversial and the semantic ambiguities can still cause confusion.

A general consensus is reached only for the definition of complicated versus non-complicated pyelonephritis: the former term refers to the presence of systemic (such as HIV positivity, diabetes, neoplasia or collagen disease) or anatomical (such as active stone disease, obstruction, reflux nephropathy) predisposing factors, the latter term to their absence [1-4].

As Talner and co-workers underlined over 15 years ago, there is no agreement on the terminology of acute pyelonephritis and, as a consequence, on diagnostic definitions [5]. Indeed there are two distinct and legitimate definitions, involving different diagnostic approaches. According to the "classical" one, reported in almost all textbooks of medicine, the definition is pathological, requiring the demonstration by imaging techniques or renal biopsy involvement (only rarely performed, limited to kidney transplants) of the renal parenchyma [5,6]. According to this definition, upper urinary tract infections (UTI) can be classified as pyelonephritis (with evidence of parenchymal involvement) or pyelitis (without evidence of parenchymal involvement at imaging). Only the pathological approach, based on imaging techniques, is apt to identify the presence of kidney scars. This approach is often followed in children, as the pediatric literature usually underlines the importance of renal scars as a potential cause of progressive kidney damage and hypertension or of pre-eclampsia later in life [6-11]. Nevertheless the issue is controversial and long-term effects of kidney scars have yet to be fully demonstrated [12-14].

The second definition is clinical only. Under the pressure of cost constraints, most Authors in the last decade have preferred (at least in adults) a clinical definition based on a classic tetrad of high fever, costovertebral angle tenderness, signs or symptoms of lower urinary tract infection and positive urinary cultures. This approach has the advantage of simplicity and lower costs but is unable to discriminate between the different forms of upper urinary tract infections (upper UTI) [6,15-17].

The therapy of upper urinary tract infections is likewise controversial, possibly because of the different prevalence of severe parenchymal lesions in the different series. General agreement is reached only on the potential danger of kidney abscesses, requiring hospitalization and long-term antibiotic treatment [6,15-17].

In this context, there are at least two good reasons to reconsider acute pyelonephritis one century after the pivotal description by Tiemlich and a decade after the K-DOQI re-definition of chronic kidney disease (CKD) [1,18].

The first is the re-definition by the K-DOQI guidelines of CKD as any persistent alteration of the morphology of the kidneys or of kidney function. Thus kidney scars define the presence of CKD, suggesting that the therapeutic goals should include their avoidance, along with the prevention of sepsis and renal abscesses [18].

The second reason regards the impressive improvements of imaging in kidney infections, allowing the identification of small lesions and the distinction between healing, fibrotic and edematous (presumably active) infectious foci [19-30]. However, despite the improvement in diagnostic tools, very few recent studies have investigated the relationship between clinical and imaging presentation in adults. Our previous studies suggested a relationship between the presence of renal abscesses at diagnosis and the development of kidney scars. They also indicated, in a cohort of cases referred to the Emergency Room, that only imaging data could distinguish between upper urinary tract infections with and without parenchymal involvement [31-33].

The aim of the present prospective study (June 2005-December 2009) was to analyze the clinical and imaging presentation of all patients hospitalized in our setting, in which diagnosis of APN was made according to imaging criteria.

Our goal was to test the relationship between the clinical-laboratory data at presentation and the imaging data. We reasoned that a strong correlation would allow a simpler, more rapid identification of the cases with the severest kidney involvement (abscessed and/or multiple lesions), requiring hospitalization and long-term therapy, while the lack of correlation would support the wide use of imaging techniques.

## Methods

### Study setting and main diagnostic pathway

All consecutive patients hospitalized for acute pyelonephritis in the Urology and Nephrology, Internal Medicine and Emergency Medicine wards of the hospital san Luigi Gonzaga in the period June 2005-December 2009 were included in the study. All patients were referred to the Nephrology Unit during hospitalization for the planning of subsequent follow-up and were followed in the Nephrology Unit at least until clinical and radiological healing.

Data were gathered prospectively, starting from the first clinical visit to the Nephrology Unit.

The san Luigi Gonzaga Hospital is a 300-bed university hospital (350, including day-hospital beds); the geographic area covers approximately 100,000 inhabitants. All patients were hospitalized from the hospital Emergency Room (ER); the ER performed over 41,000 visits per year in the first period (May 2005-May 2006) and over 55,000 per year in the last one (January 2009-December 2009).

### Clinical definitions

The diagnosis of APN was made by the ER physicians; diagnosis was based on the presence of at least one of the three main symptoms (fever, costovertebral angle tenderness or pain, recent or present UTI) and at least one sign of systemic infection (high WBC or C-reactive protein). The presence of leukocytes or nitrites was an ancillary criterion supporting the presence of UTI. The presence of shivers, routinely evaluated in the Emergency Room as part of the patients' work-up, was not uniformly recorded and was not considered as sensitive enough to be added to the selection criteria.

The diagnosis of present or recent UTI was mainly based on the clinical history and on the clinical symptoms; in fact, in our setting, patients usually start an empirical therapy (either prescribed by the family physician or self-prescribed) before undergoing urinary tests, in particular if they are prone to develop UTI. This is also the reason why, in our opinion, the prevalence of positive urinary cultures was relatively low.

All patients with a clinical picture suggestive of APN underwent abdominal ultrasound, whenever possible in the ER, and were hospitalized. In patients hospitalized for fever of unknown origin, in which the diagnosis was made during hospitalization, ultrasound was performed subsequently.

In the presence of systemic predisposing factors (any factor affecting the immune response, including diabetes, collagen diseases, chemotherapy, HIV positivity, neuromuscular diseases) and/or of anatomical predisposing factors (any factors causing obstruction, including active stone disease, prostatic hypertrophy, kidney malformations, polycystic kidney disease and indwelling catheters) the APN was considered as "complicated" or secondary and the patients were not included in the present analysis. Regarding the diagnostic pathway, in the presence of anatomical predisposing factors the most widely employed technique was CT scan, better in defining the urinary tract and superior in the definition of stone disease. Patients with complicated pyelonephritis were followed by the urological team and were not included in the analysis. Patients with systemic predisposing factors (such as diabetes or neoplasia) were followed by our group but were not included in the analysis, as the predisposing factors could affect both presentation and follow-up. Pregnant patients were also excluded. History of kidney stones was recorded, and APN was considered as non-complicated in the absence of kidney stones at imaging at diagnosis or of a history of having passed a kidney stone in the previous month. The only morphological exception was the presence of kidney scars presumably due to previous APN episodes, on the basis of the spatial relationship with the excretory system, at the first imaging.

Consent for publication and for anonymous management of data was obtained either at hospitalization or at first access in the day-hospital. Prof. Alberto Angeli from the Ethical Committee of the University of san Luigi Gonzaga assessed the study and confirmed that ethical approval was not needed since the study deals with a non-invasive diagnostic tool used according to its standard indications in a specific subset of patients, for which previous indications of the literature are available. Furthermore the study analyzes the results obtained in clinical practice and no test was performed for the sake of the study. Approval for the indication of each specific imaging test is a part of the routine controls of the Magnetic Resonance or Computerized Tomography experts, who ask for informed consent for each test and, in each case, control for the coherence of prescription of the analysis.

### Imaging criteria

The diagnosis of pyelonephritis required demonstration of involvement of the kidney parenchyma. In cases with clinical suspicion of APN, in which no predisposing condition was found at ultrasounds, a "second-line" imaging test was performed. Since patients with non-complicated APN are mostly women of childbearing age, MR was chosen as the preferred imaging technique and CT scan or scintigraphy was performed only in the case of contraindications or logistical problems (long wait before availability of MR scan). As a rule, imaging data were obtained within 3 days after hospitalization.

In view of the low sensitivity of ultrasounds for the presence of parenchymal lesions and the high sensitivity for obstructive lesions, the ultrasound imaging was used to identify the presence of anatomical predisposing factors.

At the MR scan, the diagnosis of APN requires the demonstration of the following: in the basal imaging test, the presence of minor alteration with non-homogeneous hypointensity in T1-weighted sequences and hyperintensity in T2-weighted sequences. After the injection of contrast medium (Gadolinium), in T1-weighted sequences, reduction of parenchymal contrast enhancement in the affected area is observed. Abscessed areas are defined by a peripheral halo enhancement after contrast medium.

Diffusion (DWI) sequences were also studied. APN is characterized by areas of reduction of the diffusivity of the water molecules (hyperintense in sequences b = 600); however the finding is less specific [28].

At the CT scan, the diagnosis of APN requires the demonstration of the following alterations after contrast medium: decreased enhancement with a "striped" effect of the renal parenchyma; the triangular shape originally described (lobar nephronia) in the area of decreased perfusion is typical, but not required for diagnosis.

Kidney abscesses are defined by the presence of a hyperintense peripheral border, with a marked hypodensity of the lesion (necrosis), after contrast medium.

Ancillary signs of APN, both at CT and MR scans, are: kidney swelling, perinephric inflammatory fluid and stranding of the perinephric fat and occasionally a minimal dilation of the urinary tract [28].

At DMSA-scintigraphy, the diagnosis of APN requires the demonstration of areas of altered perfusion; the definition of kidney abscesses is not possible by this technique.

### Data collection and analysis

The following general data were collected: age, sex, parity, country of origin, previous UTI, previous history of pyelonephritis, previous stone disease. By definition, patients with kidney stones were not included. At referral, the following clinical data were collected: fever (presence, considered as > 37°C; maximum level on the day of hospitalization), costovertebral angle-tenderness, lower UTI symptoms, other symptoms; antibiotic therapy within the last 72 hours; other therapies.

Laboratory data were obtained by standard laboratory methods. The following data, obtained in the Emergency Room at hospitalization, were considered: serum creatinine, CRP, blood cell counts, urinary culture (available in all cases), hemocultures and results of dipstick urinalysis were also recorded when available.

On the basis of their radiological appearance, the lesions were dichotomized in "simple" versus abscessed (at least one abscessed lesion) and in single versus multiple. Further imaging parameters were unilateral versus bilateral involvement and presence of kidney scars at diagnosis.

**Figure 1:**
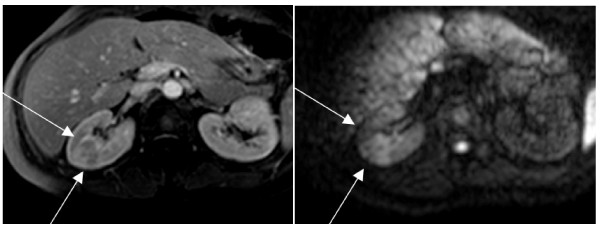
**Large simple lesion: a) T1 weighted sequence showing a large pyelonephritis focus in the right kidney; b) same area in diffusion: the large cuneiform lesion is clearly evident due to the intense edema**.

**Figure 2:**
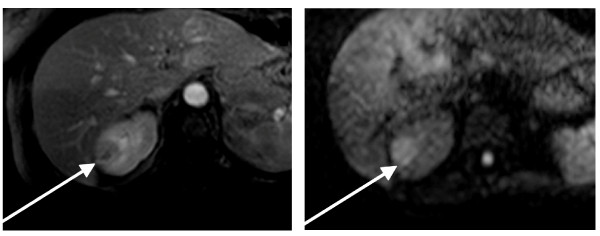
**(same patient as in figure 1)large abscessed lesion: a9 T1 weighted late contrast sequence showing a large abscessed focus in the right kidney; b) same area at diffusion**. The round shape is clearly evident in both sequences.

Informed consent for publication of the clinical data in anonymous form was obtained from all patients at the first nephrological control. Further specific informed consent was obtained from the patient whose images are depicted in Figures 1 and 2.

### Statistical analysis

Descriptive analysis was performed as appropriate (mean and standard deviation for parametric data and median and range for non-parametric data). Student's t-test, chi-square test and ANOVA were used for comparisons between groups. Significance was set at < 0.05.

Univariate odds ratios and multivariate regressions were calculated in SPSS (version 17.0). Logistic regression was performed considering the type of lesion (abscessed lesion) or the number of lesions (multiple lesions) as outcomes, with respect to the main clinical data (age, fever, costovertebral tenderness, lower urinary tract symptoms, duration of symptoms and their combination), presence of renal scars and C-reactive protein as a marker of inflammation. Continuous data were dichotomized at the median.

## Results

### Baseline data

In the period June 2005-December 2009, 119 patients with an imaging-confirmed diagnosis of acute non-complicated APN were hospitalized in our setting. The incidence was stable throughout the period: overall 2.2 cases/month (2.3 in 2005 and 2.6 in 2009). The referral area of the university hospital includes approximately 100,000 inhabitants; thus, the incidence corresponds to roughly 265 cases per year per million population (or 2.65 per 10,000 inhabitants, as the incidence of APN is often reported). The prevalence of the different types of upper UTI was assessed in a sample of 85 cases hospitalized in the Emergency department in 2008-2009 with the diagnosis of "upper UTI". In 6 cases the final diagnosis was of a different infection. In the other 79 cases, there were 33 non-complicated APN (42%), 32 complicated APN (with predisposing factors) (40%), and 14 upper UTI without radiological signs of parenchymal involvement (18%).

In keeping with the diagnosis of non-complicated (or uncomplicated) APN, all patients were females with normal genitourinary tract, with a median age of 32 years and a wide age range (15-72 years). A history of kidney stones was present in 19/119 patients (16%); none had active stone disease according to the definition of "non-complicated" pyelonephritis. Forty-nine patients had had at last one pregnancy (41%); 21 (17.7%) were taking anti-progestagens; a history of frequent urinary tract infections was reported by about half of the cases (table 1).

**Table 1:** Baseline data of the studied population (clinical history)

Patient features	Single lesions	Multiple lesions	P	Simple lesions	Abscessed Lesions	p	All cases
**N**	24	95	-	72	47	-	119

**Age**	28 (15-72)	33 (15-68)	0.38*	30.5 (15-72)	34 (16-63)	0.80*	32 (15-72)

**History of frequent UTI**	14 (58.3%)	41 (43.6%)	0.29^	38 (52.8%)	17 (36.2%)	0.11^	55 (46.2%)

**Previous scars (APN)**	2 (8.3%)	16 (16.8%)	0.47^	13 (18.1%)	5 (10.6%)	0.4^	18 (15.1%)

**History of stone disease (§)**	5 (20.8%)	14 (14.7%)	0.68^	**16** **(22.2%)**	**3** **(6.4%)**	**0.04^**	19 (16%)

**Previous pregnancy**	9 (37.5%)	40 (42.1%)	0.69^	31 (43.1%)	18 (38.3%)	0.29^	49 (41.2%)

**Birth control pills**	5 (20.8%)	21 (22.1%)	1^	15 (20.8%)	11 (23.4%)	0.92^	26 (21.8%)

* Mann-Whitney U test

^ Pearson's chi-square test

§ By definition, patients with active stone disease were excluded.

### Imaging at diagnosis

By definition, diagnosis was made by a second-line imaging technique. MR was performed in 105 cases, CT scan in 11, while renal scintigraphy was the second-line test in 3 patients with contraindication to both CR and MR (claustrophobia, multiple allergies). In these 3 cases, the lesions were considered non-abscessed on the basis of the negativity of ultrasound, sensitive to the presence of abscessed lesions.

The severity of the lesion was not uniform; "simple" non-abscessed lesions only were present in 72 patients (60.5%) while at least one abscessed lesion was present in 47 patients (39.5%) (table 1, Figure 1, 2). About 20% of the patients (24 cases) had a single renal lesion. Lesions were multiple in 95 cases (79.8%) and bilateral in 16 patients (13.5%).

In 18/119 patients (15.1%) one or more renal scars were detected at the time of diagnosis. They were considered a sign of previous APN. However a previous APN episode was reported in only 3 cases.

There was no difference in the clinical history between patients with single or multiple kidney lesions. In the univariate comparison of patients with non-abscessed versus abscessed lesions, the only statistically significant difference was the higher prevalence of previous stone diseases in the patients with "simple" lesions (table 1).

### Clinical-laboratory picture at presentation

According to the diagnostic criteria, all patients displayed at least one of the hallmarks of the classical presentation of APN (high fever, costovertebral pain and/or tenderness, signs or history of recent UTI), plus at least one sign of infection/inflammation (high CRP level, increase in WBCs) (table 2). However, the three symptoms together (high fever, costovertebral pain and/or tenderness, signs or history of recent UTI) were present in only about one third of the cases (42 patients; 35.3%). The full-blown picture (the "clinical tetrad" including the main three symptoms mentioned above, plus positive urinary cultures) was present in only 13 cases (11%).

**Table 2:** Main clinical and biochemical data at presentation

	Single lesions	Multiple lesions	p	Simple lesions	Abscessed lesions	p	All cases
SCr (mg/dL)	1.03 ± 0.43	0.89 ± 0.28	0.07°	0.95 ± 0.37	0.89 ± 0.22	0.26°	0.92 ± 0.32

CRP (mg/dL)	10.3 (1.3-39)	12.5 (0.2-36)	0.41*	10.6 (0.3-39)	13.3 (0.2-31)	0.14*	12.1 (0.2-39)

WBC (n/mm3)	13042 ± 4128	12397 ± 4139	0.49°	12103 ± 4185	13194 ± 3990	0.16°	12528 ± 4127

Positive urinary cultures	12.5%	26.3%	0.25^	19.4%	30%	0.28^	23.5%

**Positive hemocultures**	**0%**	**15.8%**	**0.004^**	**16.7%**	**6.4%**	**0.014^**	12.6%

Fever	39 (36-40)	39 (36-41)	0.12*	39 (36-40)	39 (36-41)	0.17*	39 (36-41)

Flank pain - tenderness	87.5%	88.4%	1^	84.7%	93.6%	0.24^	88.2%

Lower urinary tract symptoms	62.5%	43.2%	0.14^	41.7%	55.3%	0.20^	47.1%

Antibiotic treatment in the last 3 days	54%	39%	0.31^	43.1%	40.4%	0.33^	42%

Time between symptoms and diagnosis	3 (1-14)	3 (1-30)	0.54*	3 (1-30)	3 (1-20)	0.36*	3 (1-30)

**Tetrad (fever, pain, lower UTI, positive cultures)**	8.3%	11.7%	0.92^	**5.6%**	**19.6%**	**0.039**^	11%

Eligible for a trial (tetrad and no recent antibiotic therapy)	4.2%	9.5%	0.67^	4.2%	14.9%	0.08^	8.4%

UTI: Urinary tract infection

*Mann-Whitney U test

°Student's t-test

^Pearson's chi-square test

The median interval between the first symptoms and referral to the ER was 3 days (3 or more days in 61%), in keeping with a relatively late referral in our setting where patients are routinely assessed by the family physician before hospital referral.

Urinary cultures were positive in 23.5% of the patients. Antibiotic therapy in the last three days was reported at admission by 50 patients (42%). None of the main clinical features at presentation (lumbar pain, lower urinary tract symptoms, fever) showed a significant difference between patients with positive or negative urinary cultures; however there was marginal significance for previous antibiotic therapy (p = 0.051). There was no difference in the interval from onset of symptoms to referral between patients with positive or negative urinary cultures; however the range was wider in the latter (1-30 days versus 1-7 days), suggesting that a long interval between symptoms and referral may have contributed to the low prevalence of positive urinary cultures, at least in selected patients (table 2).

All the biochemical parameters were widely scattered. None of the main clinical and laboratory data at referral discriminated the presence of multiple lesions or the presence of at least one abscessed lesion. The only significant differences at presentation were a higher prevalence of positive urinary cultures in multiple and non-abscessed lesions and a higher incidence of the full-blown tetrad in patients with abscessed lesions (table 2).

#### Multivariate analysis

Tables 3 and 4 report the results of the multivariate analysis of the outcomes of multiple lesions (versus single) and abscessed lesions (versus non-abscessed lesions). For the outcome multiple versus simple lesions, statistical significance was reached for the combined variable of positive urinary culture and/or positive hemoculture (Odds Ratio of about 4) (table 3). Conversely, no biochemical or clinical marker at presentation showed a significant correlation with the presence of abscessed lesions at diagnosis (table 4).

**Table 3:** Multivariate logistic regression: risk of multiple lesions

N = 119	Number in Group	**Multiple lesions** **(N = 95)**	%	OR - UNI	CI 95% - UNI	OR - MULTI^1^	CI MULTI
Age ≤ 32 years	62	47	75.8%	1			

Age > 32 years	57	48	84.2%	1.702	0.679-4.267	1.699	0.620-4.653

Scars at diagnosis NO	101	79	78.2%	1			

Scars at diagnosis YES	18	16	88.9%	2.228	0.476-10.434	1.81	0.349-9.383

CRP < 12 mg/dL	58	45	77.6%	1			

CRP > = 12 mg/dL	58	47	81.0%	1.234	0.501-3.039	1.151	0.425-3.117

Negative urinary cultures or hemocultures	81	60	74.1%	1			

**Positive urinary cultures and/or hemocultures**	**38**	**35**	**92.1%**	**4.083**	**1.136-14.679**	**4.204**	**1.139-15.515**

Presence of the 3 main symptoms	77	65	84.4%	1			

Absence of at least one of the 3 main symptoms	42	30	71.4%	0.462	0.186-1.146	0.437	0.168-1.13

CRP (C-reactive protein); Age: dichotomized at the median. Three main symptoms: fever; lumbar pain-tenderness; symptoms of lower UTI

**Table 4:** Multivariate logistic regression: risk of abscessed lesions

N = 119	Number in Group	**Abscessed lesions (N = 47)**	%	OR - UNI	CI 95% - UNI	OR - MULTI^1^	CI MULTI
Age ≤ 32 years	62	23	37.1%	1			

Age > 32 years	57	24	42.1%	1.233	0.591-2.575	1.003	0.442-2.276

Scars at diagnosis NO	101	42	41.6%	1			

Scars at diagnosis YES	18	5	27.8%	0.540	0.179-1.631	0.732	0.222-2.421

CRP < 12 mg/dL	58	17	29.3%	1			

CRP > = 12 mg/dL	58	27	46.6%	2.101	0.977-4.516	2.095	0.921-4.767

Negative urinary cultures or hemocultures	81	32	39.5%	1			

Positive urinary cultures and/or hemocultures	38	15	39.5%	0.999	0.454-2.197	1.010	0.437-2.335

Presence of the 3 main symptoms	77	26	33.8%	1			

Absence of at least one of the 3 main symptoms	42	21	50.0%	1.962	0.911-4.226	2.080	0.932-4.641

CRP (C-reactive protein); Age: dichotomized at the median. Three main symptoms: fever; lumbar pain-tenderness; symptoms of lower UTI

## Discussion

The present study was performed in a setting in which patients with a clinical suspicion of "non-complicated" APN are routinely hospitalized and undergo an extensive imaging work-up (ultrasound and either MR or CT scans) [31-33].

In two recent surveys in the USA and Korea, the incidence of APN was reported as 20-35 new cases per year per 10,000 inhabitants [34-37]. Thus the gross incidence was about 10 times higher than in our study (about 2.5 new cases per year per 10,000 inhabitants). However it is difficult to compare the incidence data in our study with those in the literature, for two main reasons. Firstly, the definition of APN rests on different bases. Imaging data are not included in the diagnostic work-up in the USA and Korea but are required for diagnosis in our setting. Secondly, in the absence of an imaging work-up, the incidence data in the USA and Korea include both non-complicated and complicated pyelonephritis while our study was limited to the former category. In our setting the ratio between "complicated or secondary" and "non-complicated or primary" APN is approximately 3:1 in the Emergency Room (ER), a finding that could at least partly explain the difference.

Unlike other settings, Italian ERs are usually the second referral point after the family physician; both family physicians and ERs are provided by the national health care system and are free of charge. An efficient filter by family physicians could be postulated on the basis of the 42% prevalence of antibiotic treatment in the last 3 days and of a median time of 3 days between the onset of symptoms and hospitalization.

In this context the present study was planned with the aim of describing the clinical presentation of imaging-confirmed APN. From the practical point of view we wanted to identify clinical or biochemical markers of severity of the kidney lesions (multifocal and abscessed lesions) to test the alternatives to our imaging-guided diagnostic and therapeutic approach.

The key feature of our study is the high prevalence of severe lesions: multiple lesions were present in 79.8% of the patients and abscessed lesions in 39.5%.

In spite of the severity of the lesions we found a high prevalence of incomplete clinical presentations, lacking the full-blown syndrome classically described as high fever, signs or history of recent lower UTI, costovertebral flank pain or tenderness and positive urinary cultures (table 2). The three symptoms were present at hospitalization in about one third of cases and lower urinary tract symptoms were present in about half of the patients (47%). These clinical hallmarks are usually included in the enrollment criteria for clinical trials for APN, together with positive urinary cultures and no recent antibiotic therapy [6]. According to these criteria, only about 10% of our patients would have been eligible for a recent clinical trial on APN (table 2).

The frequent negativity of the urinary cultures deserves further comment. About half of the cases were treated with antibiotics but the difference between cases with positive and negative cultures was only marginally significant (p = 0.051). The relatively long interval between onset of symptoms and hospitalization and the frequent lack of lower urinary tract symptoms suggest alternative and adjunctive explanations.

Interestingly, a similar prevalence was found in a previous study, performed in a different setting (the largest University Hospital of the Region) in 2004-2006, in which the prevalence of positive urinary cultures was even lower (20.7%), albeit with a higher recall of antibiotic therapy (95%) [32]. This observation may indicate a "referral" bias (patients are usually referred to the emergency room after a few days of empirical therapy), more than a laboratory bias.

To further assess this point, we planned an analysis in the Emergency room to test the hypothesis that, in some cases, cultures may have been performed after the first dose of intravenous antibiotics (as may occur overnight or during the weekend, when urine sampling may be erroneously postponed in a crowded ER). The analysis is still ongoing; however even if the sampling may have been biased in some cases, this finding draws attention to the fact that urinary cultures can become negative after a single antibiotic dose, even in the presence of renal abscesses or multiple lesions.

It is difficult to explain the higher prevalence of positive cultures in non-abscessed lesions. Our hypothesis is that this may be due to the fact that the bacteria are "sequestered" in abscessed lesions. However further analysis on larger cohorts is needed to confirm this apparent paradox.

The difficulty in diagnosing APN is further underlined by the fact that over 15% of the patients displayed renal scars at the first imaging test, presumably as a result of previous APN episodes; APN had been previously diagnosed in only a minority of them (3/18).

In this context and within these limits, the major finding of our study is the lack of correlation between the severity of the renal lesions and the clinical and biochemical data at presentation (table 1, table 2, table 3 table 4). In fact oligosymptomatic presentation may coexist with abscessed or multifocal lesions and none of the tested parameters discriminated the severity of parenchymal involvement. This is of particular relevance for abscessed lesions which require long-term antibiotic therapy and are at high risk for the development of kidney scars [15-17,23,25,32].

Like all clinical studies, ours has strengths and weaknesses. The main strengths are the novelty and the systematic referral pattern with a diagnostic use of second-line imaging techniques. To our knowledge, this is the largest series of adults published in the last 10 years, combining a clinical and imaging work-up of all cases of suspected APN referred to the ER of a relatively large hospital in a short time span.

The limitations are partly shared by single-center observational studies. In the absence of a control group and of epidemiological data on the world-wide incidence of "non-complicated" APN, our data are hardly comparable with the literature. The suggestion that we may be dealing with negative selection of the cases, possibly due to the extensive filter of family physicians, needs further confirmation on a larger scale and in different settings. A potential confounder is the high prevalence of negative urinary cultures, possibly reflecting the policy of empirical antibiotic treatment before hospital referral and the lack of lower urinary symptoms but also perhaps related to late testing. This issue needs further analysis, also in view of the lack of recent epidemiological data on the prevalence of negative urinary cultures in APN [15-17,33-38].

These limitations may become suggestions for further studies on the prevalence and type of parenchymal involvement and on the clinical presentation in settings with different incidence and referral patterns.

## Conclusions

The diagnosis of APN is still a challenge. At least in settings such as ours in which patients are referred to the Emergency Room after a few days of empirical treatment, the prevalence of incomplete oligosymptomatic presentations is high.

In a context of high prevalence of abscessed lesions (39.5%), severe kidney involvement coexisted with incomplete presentations. None of the clinical or biochemical markers at presentation allowed discrimination between small simple lesions and multifocal or abscessed ones. Hence imaging techniques were needed to assess the severity of kidney involvement and to plan the antibiotic therapy. The relatively high prevalence of kidney scars at presentation (15.1%), mostly with a silent clinical history, draws attention to the difficulty in diagnosing and preventing long-term sequelae of APN.

Therefore, we believe that two recommendations for clinical practice can be drawn from our experience. First, the differential diagnosis of APN should be considered in all patients presenting at least one suggestive clinical feature (fever, flank pain, lower UTI symptoms) accompanied by high inflammatory markers. Second, second-line imaging tests (MRI or CT scans) should be systematically used to define the prevalence and type of parenchymal lesions in order to tailor interventions to the specific clinical contexts.

## Competing interests

The authors declare that they have no competing interests.

## Authors' contributions

GBP, FR and BA designed the study; GBP and BA drafted the manuscript. VC and MCD participated in the management of the patients and collected the data of patients in the Nephrology setting. MS took care of data collection and control in the Emergency Room. MB took care of the statistical analysis, supported by MS. ADP and AV carried out the RMN analysis. FP supported the definition of non-complicated pyelonephritis. All authors read and approved the final manuscript.

## Pre-publication history

The pre-publication history for this paper can be accessed here:

http://www.biomedcentral.com/1471-2369/12/68/prepub
